# Peritoneal Metastases from Extraperitoneal Primary Tumors: Incidence, Treatment, and Survival from a Nationwide Database

**DOI:** 10.1007/s13193-022-01592-w

**Published:** 2022-07-20

**Authors:** Anouk Rijken, Laskarina J. K. Galanos, Jacobus W. A. Burger, Simon W. Nienhuijs, Felice N. van Erning, Ignace H. J. T. de Hingh

**Affiliations:** 1 Department of Surgery, Catharina Cancer Institute, PO Box 1350, 5602 ZA Eindhoven, Netherlands; 2 Department of Research and Development, Netherlands Comprehensive Cancer Organization, Utrecht, the Netherlands; 3 GROW - School for Oncology and Development Biology, Maastricht University, Maastricht, the Netherlands

**Keywords:** Peritoneal metastases, Extraperitoneal origin, Incidence, Treatment, Survival

## Abstract

The objective of this study was to assess the incidence, treatment, and survival of patients with synchronous peritoneal metastases (PM) from extraperitoneal primary tumors. A cohort was selected from the Netherlands Cancer Registry (NCR), in which all patients diagnosed with PM in 2017 and 2018 were screened for eligibility. The five most common primary extraperitoneal origins of PM were included for further analyses: lung, breast, urinary tract, and kidney cancer and malignant melanoma. Survival was investigated using log-rank test between different primary tumor locations. In total, 480 patients were diagnosed with synchronous PM from extraperitoneal origins. The proportion of patients with PM per extraperitoneal origin ranged between 0.1 and 1.1%, with the highest proportion in lung cancer patients. Of all patients, 234 (49%) received tumor-directed treatment and 246 (51%) received no tumor-directed treatment. Survival in patients with PM from lung, breast, urinary tract, and kidney cancer and malignant melanoma was 1.6 months, 15.7 months, 5.4 months, 3.4 months, and 2.1 months, respectively (*p* < 0.001). In this study, a small, although significant number of patients with extraperitoneal cancer developed PM. The reported survival in patients with PM ranged between 1.6 and 15.7 months. Only half of the patients with PM received tumor-directed treatment and survival in patients without tumor-directed treatment was only 1.2 months. These findings are stressing the need to explore new diagnostic tools that may enable earlier diagnosis of PM and may potentially lead to a more effective treatment.

## Introduction

The peritoneum is a common metastatic site for intraperitoneal primary tumors. As a result, most peritoneal metastases (PM) arise in patients with colorectal, ovarian, and gastric cancer [1–3]. In all these primary tumors, PM are notorious for their detrimental impact on survival. While it is known that PM can also arise from extraperitoneal origins such as breast and lung cancer, little is published on the true incidence of PM of these extraperitoneal origins [4, 5]. PM from extraperitoneal origins are rare and hence, accurate data on incidence, management, and survival is lacking.

Pancreatic tumors are also a frequent cause of PM, reflecting both the aggressiveness of pancreatic cancer and the close proximity of the pancreas to the peritoneal cavity [6]. Since the pancreas is a secondary retroperitoneal organ because of its alteration from an intraperitoneal position to an extraperitoneal position during the embryonic development, also alternative dissemination route in comparison to primary retroperitoneal organs may be present accounting for the high number of PM [7]. With this, together with the fact that we have described PM from pancreatic cancer in detail recently, pancreatic PM were not subject of the current study and this was limited to primary extraperitoneal tumors [6].

To date, treatment strategies in patients with PM are rapidly evolving, such as the use of cytoreductive surgery (CRS) with or without hyperthermic intraperitoneal chemotherapy (HIPEC) in a selected group of patients with an intra-abdominal primary tumor [8–10]. Yet, these treatments are not explored for patients with extra-abdominal PM and currently, no suitable treatment options are described in guidelines for these patients. Thereafter, more knowledge about the overall burden and current treatment strategies of PM from extraperitoneal primary tumors is designated to lead the way for future therapeutic research.

Hence, the purpose of this study is to determine the incidence of synchronous PM from primary extraperitoneal origins and to investigate the current treatments and survival of these patients.

## Methods

### Data Source

Data from the Netherlands Cancer Registry (NCR) were used for this study. Data on patient, tumor, and treatment characteristics were routinely obtained from medical records by specially trained data managers of the NCR. The specifications of primary tumor locations, location of synchronous metastases, and histologic characteristics are registered according to the International Classification of Disease for Oncology (ICD-O). Vital status of patients in this study was obtained by linking NCR data to the municipal administrative database. In this database, all deceased and emigrated inhabitants of the Netherlands are registered. For this study, follow-up was completed until January 31, 2020. No ethics approval was required for this study, since all data were anonymized.

### Study Population

All Dutch patients diagnosed with PM in 2017 and 2018 were screened for eligibility. Patients with PM from the 5 most common extraperitoneal origins (excluding the pancreas due to its secondary retroperitoneal origin) were included for analyses. Primary tumors of the following organs were selected according to the ICD-O: lung (*C34.0–C34.3*, *C34.8*, *C34.9*, *C38.4*), breast (*C50.0–C50.6*, *C50.8*, *C50.9*), skin (*C44.0–C44.9*), urinary tract (renal pelvis, ureter, bladder, and urethra; *C65.9*, *C66.9–C68.1*, *C68.8*, *C68.9*), and kidney (*C64.1–C64.4*, *C64.8*, *C64.9*). Synchronous PM were defined according to the ICD-O (*C48.0–C48.2*, *C48.8*). Only the tumor with the earliest date of diagnosis was included in patients with multiple primary tumors or, if simultaneously diagnosed, the tumor with the highest TNM stage was included. Patient and tumor characteristics included in this study were sex, age, and synchronous systemic metastases. Treatments of patients with PM were defined as follows: (1) primary tumor resection, (2) metastasectomy, (3) systemic therapy, or (4) best supportive care (BSC) only and no tumor-directed treatment, being only palliative interventions in case of symptom control such as radiotherapy, ureteral stent, or a transurethral resection of the bladder. If patients received more than one treatment, they were included in both or more treatment categories.

### Statistical Analysis

Baseline characteristics of patients with synchronous PM were analyzed according to different primary tumor locations with the *χ*^2^ test for the categorical variables and ANOVA test for the continuous variables. Age-standardized incidence rates were calculated with the (Revised) European Standardized Rate ([R]ESR) and the World Standardized Rate, representing the number of newly diagnosed patients per 100,000 inhabitants per year, standardized by age. The most common histological subtypes among patients with PM from extraperitoneal origins were explored and the different treatments were compared according to the different primary tumor locations.

Median overall survival (OS) of patients with PM from each primary tumor was compared and also median OS between different treatments was compared by using the log-rank test. Median OS was calculated from time of diagnosis until death or end of follow-up. All patients alive on January 31, 2020, were censored. All tests were two-sided and a *p* value lower than *0.05* was considered statistically significant. Analyses were performed with SAS statistical software (SAS system 9.4, SAS Institute, Cary, NC, USA).

## Results

### Study Population

**Fig. 1:**
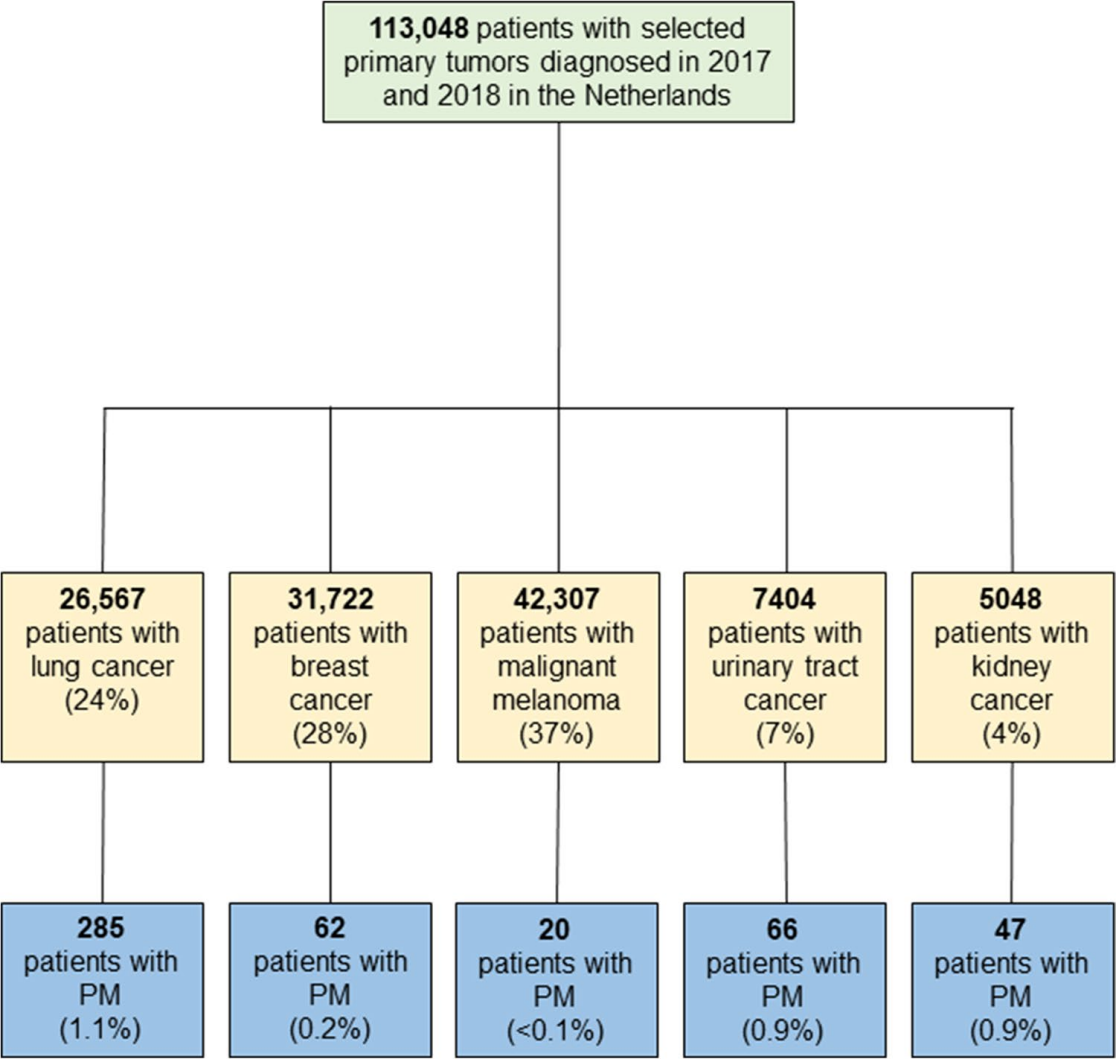
Flowchart of the study population

**Fig. 2:**
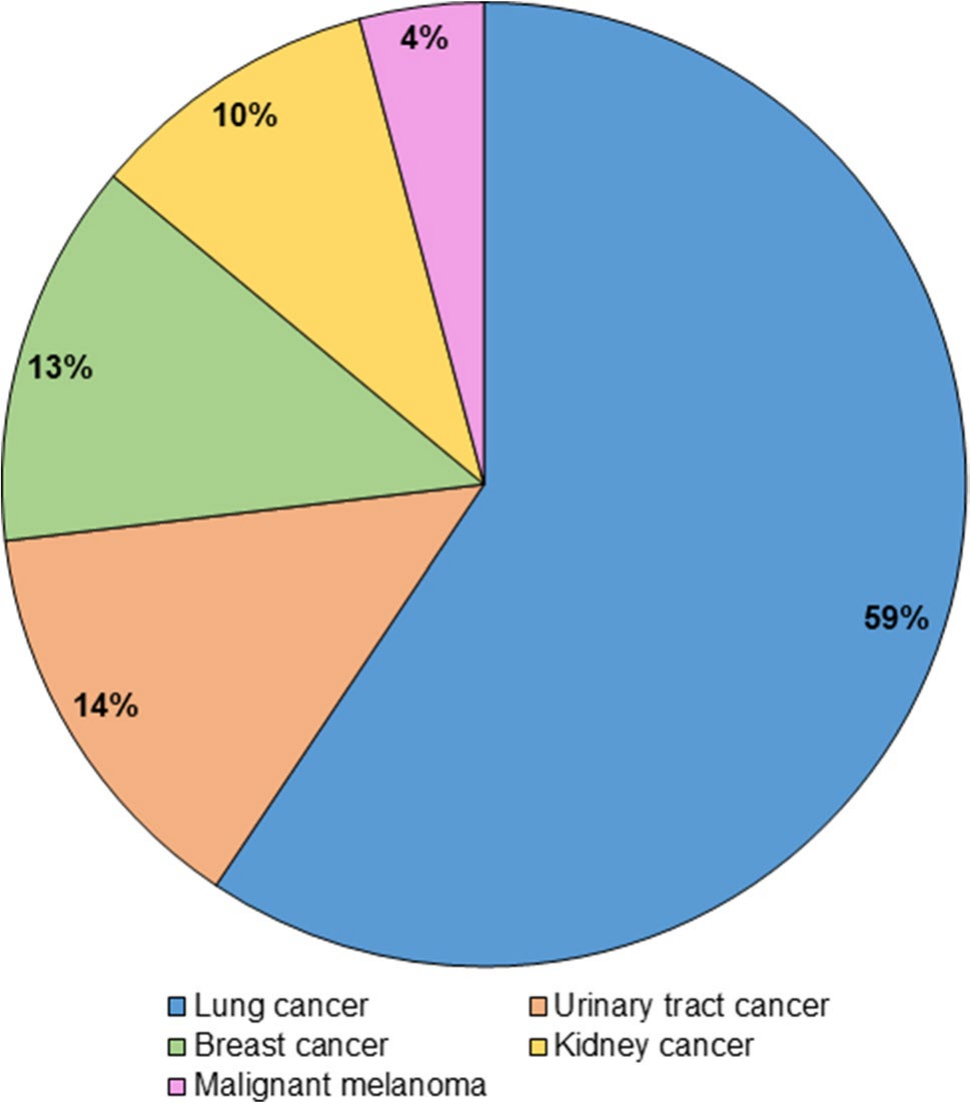
Distribution of primary tumor location in 480 carcinomas with synchronous PM from the selected extraperitoneal origins

Of all patients diagnosed in 2017 and 2018 with lung, breast, urinary tract, and kidney cancer and malignant melanoma (*n* = 113,048), 480 (0.4%) patients were diagnosed with synchronous PM. The proportions of PM within each primary tumor group varied between < 0.1 and 1.1% (Fig. 1). In all patients with PM, 285 (59%) had PM from lung cancer, 62 (13%) had PM from breast cancer, 20 (4%) had PM from malignant melanoma, 66 (14%) had PM from urinary tract cancer, and 47 (10%) had PM from kidney cancer (Fig. 2).

Of the patients with PM, 417 (87%) patients also had systemic metastases. The male sex was more frequently present in patients with PM from extraperitoneal origins except for patients with PM from breast cancer. The median age ranged between 66 and 70 years between the different primary tumors (Table 1). Table 2 shows the ESR, RESR, and WSR of patients with PM from extraperitoneal primary origins; these numbers represent the incidence of PM from extraperitoneal primary origins in the Dutch population that were corrected for the European and worldwide age distribution. The reported incidences were higher in males than in females. The WSR (0.64) is lower compared to the ESR (0.94).

**Table 1:** Characteristics of patients with peritoneal metastases from extraperitoneal origins (*n* = 480)

	Total, *n* = 480	Lung, *n* = 285	Breast, *n* = 62	Malignant melanoma, *n* = 20	Urinary tract, *n* = 66	Kidney, *n* = 47	*p* value
Sex, *n* (%)							
Male	264 (55)	173 (61)	2 (3)	15 (75)	41 (62)	33 (70)	
Female	216 (45)	112 (39)	60 (97)	5 (25)	25 (38)	14 (30)	< *0.001*
Age, median (IQR)	69 (61–76)	69 (62–75)	66 (56–76)	68 (52–75)	70 (63–81)	70 (63–77)	*0.029*
Isolated PM, *n* (%)							
Yes	63 (13)	24 (8)	7 (11)	1 (5)	27 (41)	4 (9)	
No	417 (87)	261 (92)	55 (89)	19 (95)	39 (59)	43 (91)	< *0.001*

*IQR*, interquartile range; *PM*, peritoneal metastases

**Table 2:** Age-standardized incidence rates per 100,000 inhabitants of the years 2017 and 2018 of peritoneal metastases from extraperitoneal origins

	ESR			RESR			WSR		
	Total	Male	Female	Total	Male	Female	Total	Male	Female
2017/2018	0.94	1.08	0.83	1.40	1.64	1.20	0.64	0.73	0.57

*ESR*, European Standardized Rate; *RESR*, Revised European Standardized Rate; *WSR*, world standardized rate

### Different Histological Subtypes

The most common primary lung cancer tumor morphology among the patients with PM was adenocarcinoma (*n* = 117/285; 41%), followed by small cell carcinoma (*n* = 47/285; 16%), squamous cell carcinoma (*n* = 26/285; 9%), and non-small cell carcinoma (*n* = 24/285; 8%). In breast cancer, lobular carcinoma (*n* = 29/62; 47%) followed by infiltrating ductal carcinoma (*n* = 24/62; 39%) was the most common histology subtypes among patients with PM. Malignant melanoma was the only tumor morphology in patients with PM from this origin (*n* = 20; 100%). Transitional cell carcinoma (*n* = 40/66; 61%) was the most frequent tumor morphology among patients with PM from urinary tract cancer. The most common primary kidney cancer tumor morphology among patients with PM was renal cell carcinoma (*n* = 27/47; 57%).

### Treatments in Patients with PM

Of all patients with PM from extraperitoneal primary origins (*n* = 480), 234 (49%) received tumor-directed treatment in which 24 (5%) underwent primary tumor resection, 17 (4%) underwent metastasectomy of whom 4 underwent a metastasectomy of the peritoneum, and 217 (45%) received systemic therapy. Percentages and numbers do not add up because 23 patients received more than one treatment. In 246 (51%) patients, only BSC was given and no tumor-directed treatment. The proportion of patients who underwent metastasectomy was the highest in patients with PM from kidney cancer (9%). Furthermore, the proportion of patients who received systemic therapy was the highest in patients with PM from malignant melanoma (75%) or breast cancer (81%) (Table 3).

**Table 3:** Different treatment strategies of patients with peritoneal metastases from the selected extraperitoneal tumors (*n* = 480)

	Total, *n* = 480	Lung, *n* = 285	Breast, *n* = 62	Malignant melanoma, *n* = 20	Urinary tract, *n* = 66	Kidney, *n* = 47
Primary tumor resection	24 (5)	1 (0.4)	3 (5)	10 (50)	5 (8)	5 (11)
Metastasectomy	17 (4)	9 (3)	1 (2)	1 (5)	2 (3)	4 (9)
Systemic therapy	217 (45)	111 (39)	50 (81)	15 (75)	17 (26)	24 (51)
Only best supportive care	246 (51)	168 (59)	12 (19)	1 (5)	45 (68)	20 (43)

All values are *n* (%). Percentages do not add up because 23 patients received more than one treatment

### Survival of PM

The median follow-up time for the total study population was 6.1 months. The median OS of all patients with PM was 2.4 months (interquartile range [IQR] 0.9–7.3). The median OS was 1.6 months (IQR 0.8–5.1) in patients with PM from lung cancer, 15.7 months (IQR 1.7–*not reached*) in patients with PM from breast cancer, 5.4 months (IQR 3.9–*not reached*) in patients with PM from malignant melanoma, 2.1 months (IQR 1.0–6.9) in patients with PM from urinary tract cancer, and 3.4 months (IQR 1.1–11.0) in patients with PM from kidney cancer (*p* < 0.001).

Median OS was 6.4 months (IQR 3.1–19.0) in patients who underwent tumor-directed treatment (i.e., primary tumor resection, metastasectomy, or systemic treatment) and median OS was 1.2 months (IQR 0.6–2.1) in patients who received only BSC (*p* < 0.001).

## Discussion

PM are generally known to arise from primary tumors within the peritoneal cavity such as in 6% of colorectal and in 21% of gastric cancers [1–3]. Also, cancer from the secondary retroperitoneal positioned pancreas disseminates frequently to the peritoneum (14%), most likely due to the aggressive tumor behavior, its close proximity to the peritoneal cavity, and its intraperitoneal position during embryogenesis [6]. The present study revealed that PM can also arise from other extraperitoneal malignancies as they occurred in 480 patients during the study period in the Netherlands. Lung cancer was the most frequent primary tumor origin. Furthermore, it was demonstrated that malignant melanoma, being one of the most rapidly increasing cancers in the Netherlands, also disseminates to the peritoneum albeit rarely.

In patients with intra-abdominal tumors, it is assumed that PM emerge from loco-regional tumor cell dissemination [11]. In this study, PM arise from primary tumors outside of the peritoneal cavity and the pathophysiology of peritoneal spread in these patients is not yet fully understood [4, 12]. Some experts hold the opinion that extraperitoneal PM have to be the result of hematogenous or lymphatic spread [12]. Moreover, in this cohort the vast majority of patients with PM also had other synchronous systemic metastases (87%). This finding may indicate that PM from extraperitoneal primary tumor origins are the consequence of advanced metastatic disease. In contrast, in patients with urinary tract cancer, nearly half of the patients had isolated PM. This finding could be explained by loco-regional tumor dissemination rather than systemic spread due to the primary tumor location near the peritoneal cavity.

This study identified that PM was found in 1.1% of all patients with lung cancer. Some previous studies reported on patients with PM from lung cancer, where the incidence ranged from 0.4 to 2% [4, 13–16].

Of all patients with breast cancer, only 0.2% presented with synchronous PM. Limited data on incidence of PM from breast cancer is available [4, 17, 18]. Flanagan et al. reported that PM from breast cancer was the most common extra-abdominal cause of PM but did not report the proportion PM of all patients with breast cancer specifically in their study [4]. In contrast, the present study reveals that only eight of synchronous PM from the selected extraperitoneal cancers arise from breast cancer. This could be explained by the fact that Flanagan et al. reported the synchronous and metachronous PM from breast cancer, and in a small cohort, it was found that metachronous gastrointestinal metastases frequently occur after curative treatment for breast cancer [19, 20]. Thus, the reported incidence of PM in the present study is likely to be an underestimation of the true incidence since no metachronous PM were included.

Besides a few case reports on PM from malignant melanoma, Flanagan et al. reported on malignant melanoma as the third most common cause of extra-abdominal cancer to metastasize to the peritoneum. In our study, malignant melanoma was the fifth most common cause to disseminate to the peritoneum which seems smaller. However, this can be explained since urinary tract and kidney cancer were also included in the present study as opposed to the study of Flanagan [4, 21, 22].

In literature, only a small cohort study and a case report are available on PM from urinary tract and kidney cancer and they did not report on incidence numbers [23, 24]. In this study, PM from urinary tract and kidney cancer were quite relevant as they ranked as the second and fourth most common extraperitoneal cancer causes of PM respectively.

In the present study, presented age-standardized incidences on PM from extraperitoneal primary tumors reveal that the WSRs are lower than the ESR and RESR (0.64 vs. 0.94 and 1.40). This difference in incidence rates could be explained by the fact that the worldwide life expectancy is lower than in Europe. People at older age are at a higher risk to be diagnosed with a malignancy and thus potentially to develop PM [25]. Furthermore, a sex-related difference is seen in the ERS, RESR, and WSR, with the female sex having lower incidence rates for PM than the male sex. This phenomenon was also described in studies on colorectal, gastric, and pancreatic PM [1, 2, 6].

As shown in this study, survival of patients with PM from extraperitoneal origin is generally very poor and is typically only a few months. An exception appears to be PM from breast cancer with a median OS of 15.7 months. The difference in OS could be explained by the high number of patients with PM from breast cancer that could be treated with tumor-directed therapy and a smaller proportion of patients that only received BSC compared to the other primary tumor origins. With the introduction of anti-hormonal and targeted therapies, palliative treatment options have expanded for patients with advanced or metastatic breast cancer and consequently improved OS of these patients [26–28]. No specific data on systemic therapy for PM from breast cancer is yet available; therefore, future research investigating the effect of targeted therapies for these patients is designated.

This study has some limitations. The present study reported only information on synchronous PM and did not contain any information on metachronous PM. Moreover, the detection of PM with radiological imaging techniques is known to be difficult and a diagnostic laparoscopy is usually not performed in the standard work-up of these tumors [12]. Therefore, the reported incidence of PM is most likely to be an underestimation of the true incidence of PM in these primary tumor origins. However, the present study contained nationwide data from the NCR which is characterized by a high registration coverage of more than 95% of all diagnosed cancers [29].

In conclusion, this study provides further insights into the incidence, treatment, and survival of synchronous PM from extraperitoneal primary tumors and therefore may serve as a basis for future research on PM from extraperitoneal primary origins. The present study showed that a small, although significant number of patients with extraperitoneal cancer developed PM. The reported survival in patients with PM regarding the selected primary tumors ranged between 1.6 and 15.7 months. Only half of the patients received tumor-directed treatment and survival in patients without tumor-directed treatment was only 1.2 months. These findings are stressing the need to explore new diagnostic tools that may enable earlier diagnosis of PM and may potentially lead to a more effective treatment.
